# Efficacy of Gasserian Ganglion High-Voltage, Long-Duration Pulsed Radiofrequency Combined With Block on Acute/Subacute Zoster-Related Trigeminal Neuralgia

**DOI:** 10.1155/2024/1992483

**Published:** 2024-09-19

**Authors:** Yinghao Song, Ziheng Yu, Jingjing Guan, Haisheng Wu, Qiaoling Liu, Min Yuan, Xinzhi Cheng, Bingyu Ling

**Affiliations:** ^1^ Department of Pain Northern Jiangsu People's Hospital Affiliated to Yangzhou University, Yangzhou, Jiangsu, China; ^2^ Department of Emergency Northern Jiangsu People's Hospital Affiliated to Yangzhou University, Yangzhou, Jiangsu, China

**Keywords:** acute, block, Gasserian ganglion, high-voltage, long-duration, pulsed radiofrequency, subacute, trigeminal neuralgia

## Abstract

**Background:** Trigeminal postherpetic neuralgia (TPHN) is a severe chronic pain that can lead to various socioeconomic consequences. Therefore, it is necessary to explore optimal treatment options for acute/subacute herpes zoster (HZ)–related trigeminal neuralgia and prevent the further development of TPHN. High-voltage, long-duration pulsed radiofrequency (HL-PRF) of the Gasserian ganglion is a new surgical intervention used to treat PHN. A ganglion block has been reported to possess anti-inflammatory effects and potential analgesic benefits.

**Methods:** We included 83 patients with HZ-related acute/subacute trigeminal neuralgia admitted from January 1, 2021, to June 1, 2023, and received Gasserian ganglion HL-PRF combined with block. A 6-month follow-up was conducted, including Numerical Rating Scale (NRS) scores, Pittsburgh Sleep Quality Index (PSQI), the incidence of TPHN, the dosage of anticonvulsants and analgesics, efficacy, and adverse events.

**Results:** All patients showed a significant decrease in postoperative NRS scores (*p* < 0.05). The NRS scores of the acute HZ group were consistently lower than those of the subacute HZ group at different time points (*p* < 0.01). The overall incidence of TPHN from the onset of HZ to 12 weeks is 21.68%. The incidence of TPHN in the acute phase group was 12.77%, significantly lower than the 33.33% in the subacute phase group (*p*=0.024). The effective rate was 74.7% in all patients, at 3 months after the treatment. The effective rate was 82.98% in the acute phase group and 63.89% in the subacute phase group, showing a statistically significant difference (*p*=0.047). The PSQI score of the acute group was consistently lower than that of the subacute group (*p* < 0.01). The dosage of analgesics and anticonvulsants used in the acute HZ group was lower than that in the subacute group (*p* < 0.01). All patients did not experience serious adverse reactions.

**Conclusions:** Gasserian ganglion HL-PRF combined with block can be an effective and safe technique to relieve the pain of acute/subacute zoster-related trigeminal neuralgia and prevent the incidence of TPHN.

## 1. Introduction

Herpes zoster (HZ), also known as shingles, is a skin disease caused by the reactivation of the varicella-zoster virus (VZV) within the spinal and cranial nerves. It is characterized by persistent or paroxysmal stabbing or burning pain in the affected nerve distribution area [1, 2]. The annual incidence of HZ is approximately 3.4 cases per 1000 individuals [3]. Recent surveys suggest that the incidence of HZ may still be increasing and has more than quadrupled in the past 60 years [4]. The trigeminal nerve is one of the most common sites of HZ infection, accounting for approximately 16.3% of cases [5, 6]. Currently, trigeminal neuralgia associated with HZ is a challenging and refractory neuropathic pain condition, with a lack of fully effective treatment options and a higher likelihood of developing into trigeminal postherpetic neuralgia (TPHN), which is more difficult to treat compared to other sites [7, 8]. Acute HZ-related trigeminal neuralgia refers to facial pain occurring within 30 days after the onset of shingles, subacute HZ-related trigeminal neuralgia refers to facial pain occurring between 31 and 90 days after the onset of shingles, and TPHN is defined as facial pain persisting for more than 3 months after the onset of shingles and resulting from acute and subacute HZ-related trigeminal neuralgia, being the most common and severe type [9, 10]. This chronic and severe facial pain caused by HZ infection leads to a significant social burden and a decline in patients' quality of life [11]. Therefore, pain physicians should explore an effective approach to alleviate this severe neuropathic pain and prevent the occurrence of TPHN.

Nerve block therapy has been reported as a technique for treating HZ-related pain. By using local anesthetic to block C-nociceptive neurons and the transmission of pain stimuli, it can relieve acute HZ pain [12, 13]. Although there is controversy regarding the use of steroid hormones, steroids can alleviate acute neuron inflammation [14]. Previous studies have demonstrated that injection of local anesthetics and steroids closer to the site of inflammation results in better anti-inflammatory effects [15]. Targeted ganglion blockade during the acute phase of HZ not only helps control acute pain, hyperalgesia, and allodynia symptoms but also prevents the occurrence of PHN [16].

Pulsed radiofrequency (PRF) is a minimally invasive treatment that delivers intermittent pulse currents to the target nerves to inhibit ectopic discharge, thereby modulating nerve function and relieving neuropathic pain. So far, several systematic reviews and meta-analyses have indicated that PRF is a safe and effective measure for treating HZ neuralgia from the face to the lumbosacral region, and HL-PRF, as a new treatment option, has been gaining attention for various pathological neuralgias [17]. HL-PRF is a nondestructive treatment technique that acts on neural tissue. Compared to conventional PRF, HL-PRF delivers higher energy and generates a stronger electric field effect within the neural tissue. This strong electric field can inhibit nerve conduction, reduce the transmission of pain signals, and alleviate pain. HL-PRF has advantages over conventional PRF, as it has a wider treatment range and can simultaneously target multiple nerve branches [17], resulting in improved treatment efficacy. Furthermore, the treatment process of HL-PRF is very safe, with virtually no significant complications or nerve damage [18]. The current research suggests that HL-PRF has shown good results in the treatment of TPHN. It can significantly alleviate pain, improve quality of life, and have a long-lasting effect [19].

Both nerve blocks and PRF have their limitations, with nerve blocks having a short duration of effects [20] and PRF having a slow onset of action [21]. Therefore, combining the two can better enhance the therapeutic effect. The affected semilunar ganglion can serve as a target site for the treatment of trigeminal HZ. In order to determine and provide guidance on a specific treatment, a retrospective analysis was conducted on the analgesic effect of HL-PRF combined with nerve blocks in the treatment of acute/subacute HZ-related trigeminal neuralgia.

## 2. Materials and Methods

### 2.1. Clinical Data

This retrospective study protocol was approved by the ethics committee. The study is conducted in accordance with the ethical principles and requirements outlined in the Helsinki Declaration. Patient data are strictly confidential and analyzed anonymously. Due to the patients having signed informed consent prior to the surgery, the requirement for patient informed consent was waived by the ethics committee as there was no need to contact patients again for this study. Medical records of acute/subacute HZ-related trigeminal neuralgia patients who underwent ganglion block treatment between January 2021 and June 2023 were retrieved from the Hospital Information System (HIS).

### 2.2. Study Design and Participants

This is a single-center retrospective study without a control group. Patients who met the following criteria were included in the study: (1) patients aged ≥ 18 years; (2) patients with acute trigeminal neuralgia associated with HZ (within 30 days after zoster onset) and subacute trigeminal neuralgia associated with HZ (between 30 and 90 days after zoster onset); and (3) patients who underwent trigeminal ganglion HL-PRF combined with block after hospitalization. The exclusion criteria were as follows: (1) patients with incomplete medical records (lack of baseline data or follow-up of less than 12 weeks after the procedure).

### 2.3. Surgery Procedures

**Figure 1:**
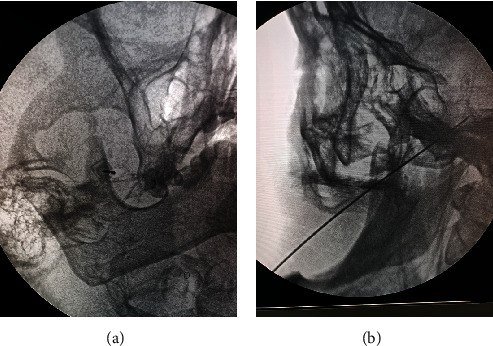
The surgical process of pulsed radiofrequency. (a) Adjust the appropriate angle of the fallopian tube to expose the foramen ovale, and puncture it into the foramen ovale with a radiofrequency needle under X-ray guidance. (b) Display the depth reached by the radiofrequency needle tip under lateral X-ray fluoroscopy.

**Figure 2:**
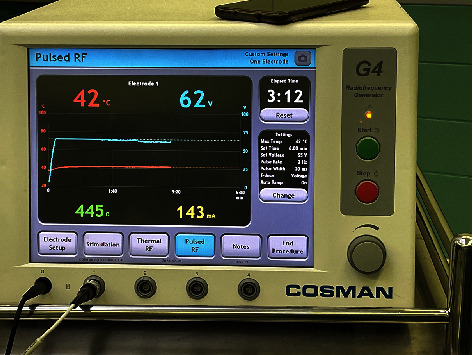
Radiofrequency instrument parameters during surgery.

All surgeries were performed by experienced pain physicians. After patients received intravenous infusion, they were brought into the operating room. Vital signs including electrocardiogram, blood pressure, and oxygen saturation were monitored, and nasal oxygen was administered. Patients were positioned supine with their head in a neutral position and a thin pillow under the neck. They were instructed to gaze straight ahead. The puncture site was located at the intersection of a vertical line passing through the lateral margin of the affected-side orbit and the horizontal line passing through the ipsilateral commissure, approximately 2-3 cm from the commissure. The Hartel approach was used for puncture throughout the procedure. A C-arm X-ray machine was rotated to expose the foramen ovale (generally, the C-arm was rotated approximately 30° toward the head end and 20° toward the unaffected side to clearly display the affected-side foramen ovale). The puncture site and surrounding skin were routinely disinfected, a sterile drape was applied, and local anesthesia was administered with 0.5% lidocaine. A 10-cm RF puncture needle was selected. Under anteroposterior fluoroscopy, the needle was advanced inward, posteriorly, and upward (Figures 1(a) and 1(b)). When it reached the foramen ovale, it was connected to the interventional RF pain treatment instrument (Cosman, USA) and switched to the sensory testing mode (50 Hz, 1.0 ms) to perform sensory testing. The patient was asked if paresthesia covered the original painful area, and the needle tip position was adjusted based on the patient's description to achieve a stimulation current < 0.3 V, which elicited corresponding facial paresthesia. PRF treatment was then performed using the PRF mode with the basic settings of 42°C, 2 Hz, 20 ms, and 900 s. The initial electric voltage was 40 V, which was gradually increased until the patients could not tolerate abnormal sensations (such as burning pain) (Figure 2). Intraoperative voltage is shown in Table 1. All patients tolerated their individual maximum voltage until the 900-second PRF treatment was terminated. Blood and cerebrospinal fluid were not drawn back, and a neuroblockade solution composed of 0.5 mL dexamethasone, 0.5 mL 2% lidocaine, and 0.5 mL normal saline was injected.

### 2.4. Data Collection

Baseline characteristics, intraoperative records, and postoperative efficacy data were obtained from the HIS medical record system. Preoperative baseline data included patient's gender, age, location of HZ, days since onset of HZ, Numerical Rating Scale (NRS) score, preoperative analgesic drug dosage, and comorbidities such as hypertension, diabetes, coronary heart disease, and stroke. Intraoperative data included surgical time, radiofrequency parameters, and intraoperative complications. Patients were followed up regularly at postoperative 1 day, 1 week, 2 weeks, 1 month, 3 months, and 6 months, as well as 3 months after the onset of HZ. Follow-up included NRS scores, Pittsburgh Sleep Quality Index (PSQI), medication usage, and dosage. Postoperative complications such as facial swelling, facial numbness, bruising, weakened chewing muscle strength, puncture site infection, or hematoma, as well as intracranial bleeding or infection, were also recorded in the HIS medical record system or through telephone follow-up. The incidence of PHN is defined as the proportion of cases with NRS scores greater than three in all cases of persistent pain 3 months after the onset of HZ. Efficacy rate was defined as the proportion of cases with a > 50% reduction in the NRS score at 12 weeks after surgery among all cases [14].

### 2.5. Statistical Analysis

The normality of demographic data was assessed using the Kolmogorov–Smirnov test. Normally distributed continuous data were presented as means ± standard deviations (SDs) and analyzed using independent *t*-tests. Non-normally distributed continuous data were presented as medians and interquartile ranges (IQRs) and analyzed using the Mann–Whitney *U*-test. Repeated-measures analysis of variance was utilized to evaluate changes in the NRS score, SF-36 score, and medication doses over time. A *p* value less than 0.05 was considered statistically significant. All data were analyzed using IBM SPSS Statistics software (version 26, IBM Inc., USA).

## 3. Results

### 3.1. Baseline Characteristics

From January 2021 to January 2023, the research center reviewed 87 cases of patients with acute/subacute trigeminal neuralgia associated with HZ who underwent HL-PRF combined block of the Gasserian ganglion. Among the 87 patients, 4 were excluded based on exclusion criteria. Therefore, we analyzed the medical records of 83 patients. Among the 83 patients, 47 cases in the acute HZ group and 36 cases in the subacute HZ group underwent HL-PRF combined block. The summarized intraoperative and baseline data of the subjects are shown in Table 1. The baseline data of the acute phase group and subacute phase group are shown in Table 2. The average time from the onset of pain to receiving the Gasserian ganglion HL-PRF combined block was 34.92 days, with 24.21 days for the acute HZ group and 48.81 days for the subacute HZ group. Gender, age, weight, affected side, affected branch, underlying diseases, preoperative NRS score, preoperative PSQI score, and preoperative medication for both groups are presented in Tables 1 and 2.

### 3.2. Primary Outcome

#### 3.2.1. NRS

**Figure 3:**
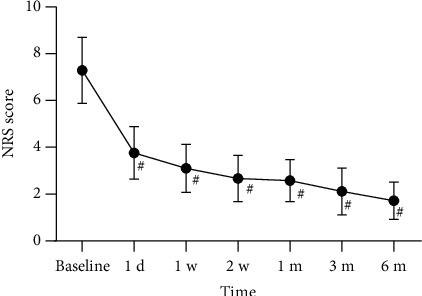
Postoperative NRS scores at each follow-up time point in all patients. Compared with baseline, ^#^*p* < 0.05.

**Figure 4:**
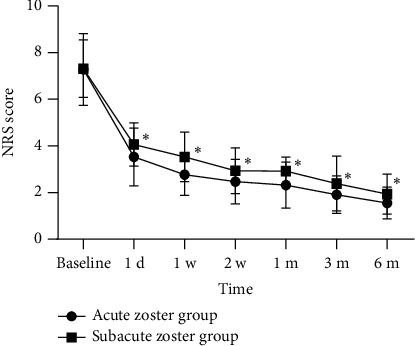
The comparison of postoperative NRS scores at each follow-up time point in two groups. Compared with the acute zoster group, ^∗^*p* < 0.01.

The preoperative NRS scores and the postoperative scores at 1 day, 1 week, 2 weeks, 1 month, 3 months, and 6 months are shown in Figure 3. All patients had a preoperative NRS score of 7.29. The postoperative NRS scores at various follow-up time points were significantly lower compared to preoperative scores. Compared to the subacute HZ group, the acute HZ group showed lower postoperative NRS scores at each follow-up time point. The differences were statistically significant (Figure 4).

### 3.3. Secondary Outcome

#### 3.3.1. Incidence of TPHN

The overall clinically significant incidence of TPHN from the onset of HZ to 12 weeks is 21.68%. In the acute phase group and the subacute phase group, there were 6 cases and 12 cases, respectively, with clinically significant PHN. The clinically significant incidence of TPHN in the acute phase group was 12.77%, significantly lower than the 33.33% in the subacute phase group (*p*=0.024).

#### 3.3.2. PSQI

**Figure 5:**
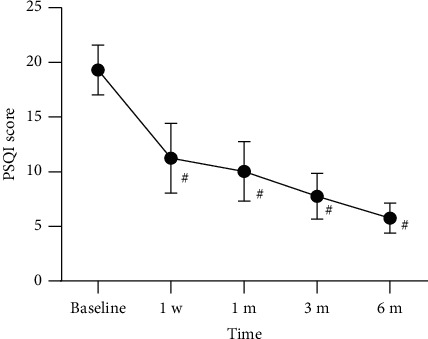
Postoperative PSQI scores at each follow-up time point in all patients. Compared with baseline, ^#^*p* < 0.05.

**Figure 6:**
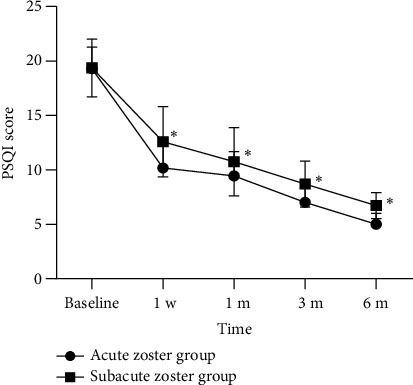
The comparison of postoperative PSQI scores at each follow-up time point in two groups. Compared with the acute zoster group, ^∗^*p* < 0.01.

The preoperative PSQI score and the postoperative score at 1 day, 1 week, 1 month, 3 months, and 6 months are shown in Figure 5. The preoperative PSQI score for all patients was 19.35. The postoperative PSQI scores at 1 day, 1 week, 1 month, 3 months, and 6 months were significantly lower compared to preoperative scores. Compared to the subacute HZ group, the acute HZ group showed lower postoperative PSQI scores at each follow-up time point, and the differences were statistically significant (Figure 6).

#### 3.3.3. Doses of Anticonvulsants and Analgesics

**Figure 7:**
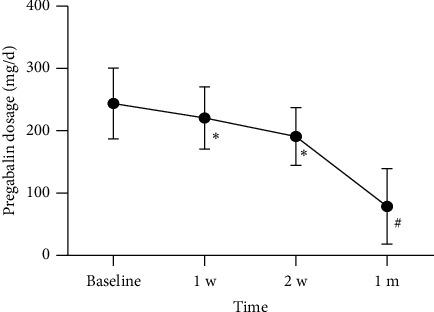
Postoperative dosage of pregabalin at each follow-up time point in all patients. Compared with baseline, ^#^*p* < 0.05 and ^∗^*p* < 0.01.

**Figure 8:**
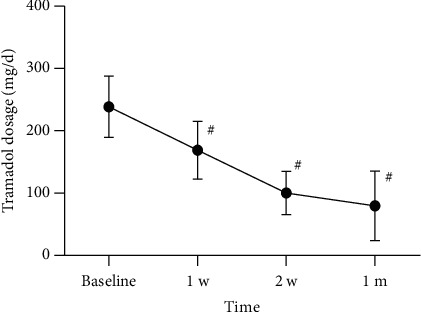
Postoperative dosage of tramadol at each follow-up time point in all patients. Compared with baseline, ^#^*p* < 0.05.

**Figure 9:**
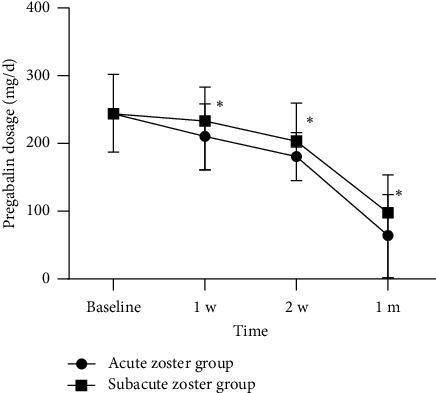
The comparison of postoperative dosage of pregabalin at each follow-up time point in two groups. Compared with the acute zoster group, ^∗^*p* < 0.01.

**Figure 10:**
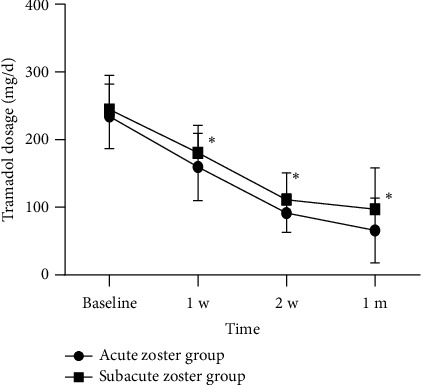
The comparison of postoperative dosage of tramadol at each follow-up time point in two groups. Compared with the acute zoster group, ^∗^*p* < 0.01.

The preoperative and postoperative doses of anticonvulsants and analgesics are shown in Figures 7 and 8. Before receiving treatment, there was no statistically significant difference in the dosage of pregabalin and tramadol between the two groups. The doses of pregabalin and tramadol continued to decrease within 1 month after surgery. However, at 7, 14, and 28 days after treatment, the dosage of pregabalin in the acute phase group was significantly lower than that in the subacute phase group (Figure 9). Similarly, at 7, 14, and 28 days after treatment, the dosage of tramadol in the acute phase group was also significantly lower than that in the subacute phase group (Figure 10).

#### 3.3.4. Effectiveness Rate

At 3 months after treatment, the proportion of patients with a 50% reduction in NRS scores was 74.7% for all patients, 82.98% for the acute HZ group, and 63.89% for the subacute HZ group. The difference between the two groups was statistically significant (*p*=0.047).

#### 3.3.5. Adverse Events

During the HL-PRF combined block treatment, there were no severe adverse reactions observed in either group, such as intracranial infection, new cranial nerve injury symptoms, or intracranial bleeding. In the acute phase group and the subacute phase group, there were three and two cases, respectively, experiencing bradycardia (heart rate < 60) during puncture of the foramen ovale. Administration of atropine (0.5 mg) restored the heart rate to normal in these cases.

## 4. Discussion

Our study retrospectively reported the use of fluoroscopy-guided HL-PRF combined with block for the treatment of 83 patients with acute/subacute HZ-related trigeminal neuralgia. Our results indicate that the HL-PRF combined block of the Gasserian ganglion is a successful therapeutic approach, with an efficacy rate of 74.7% at 3 months posttreatment and no severe complications. However, due to the lack of a control group, we must exercise caution in acknowledging the effectiveness of the treatment. Our research findings align with those of Sun et al. [14], as the NRS scores postoperatively showed significant decreases at different follow-up time points. The study suggests that Gasserian ganglion block can alleviate pain in patients with acute/subacute HZ-related trigeminal neuralgia. However, there is no significant difference in treatment efficacy between the acute phase group and the subacute phase group. However, our research revealed that the efficacy of HL-PRF combined block for HZ in the acute phase group was 82.98%, which is higher than that in the subacute phase group, and the difference is statistically significant. In contrast to previous studies [14], we chose a combination of betamethasone and lidocaine as the neuroblockade drugs instead of dexamethasone and lidocaine. Compared to dexamethasone, the local action time of betamethasone is longer, providing a more prolonged therapeutic effect. However, the optimal drug combination for the block of the Gasserian ganglion still needs further investigation in future studies.

In our study, we conducted blockade therapy following PRF on the Gasserian ganglion, and all patients experienced immediate relief of pain right after the procedure. This finding is consistent with the other study [14]. The results of this study demonstrate that the accuracy of the puncture site and the diagnosis of the source of pain significantly impact the treatment efficacy. A meta-analysis [20] also revealed that PRF targeting the Gasserian ganglion is superior to peripheral nerves in relieving pain, confirming that PRF targeting the Gasserian ganglion provides better clinical outcomes. Patients with acute/subacute HZ-related trigeminal neuralgia experienced immediate relief of pain after treatment. On the first day postoperatively, the average NRS score significantly decreased, accounting for 52.3% of all patients' preoperative NRS scores. The results demonstrate that nerve blockade can rapidly alleviate the degree of pain, compensating for the slow onset of PRF. NRS scores continued to decrease at 1 week, 2 weeks, 1 month, 3 months, and 6 months postoperatively, indicating a sustained reduction in pain levels after treatment with no recurrence.

A meta-analysis [20] found that there was no significant difference in VAS scores between the PRF and nerve block groups 1–3 days after intervention. However, PRF demonstrated better pain relief at 1, 4, and 12 weeks postintervention. The results indicate that the analgesic effect of PRF develops slowly and requires more time to reach its optimal effectiveness. Simultaneously, this finding aligns with other literature studies, suggesting that the neuroregulatory effects of PRF gradually occur and reach their maximum value around 3 months postintervention [7]. This phenomenon can be explained by the potential mechanisms of PRF. HZ-related neuralgia occurs due to persistent inflammation and nerve damage caused by the HZ virus, leading to functional changes in neurons, resulting in ectopic spontaneous discharges and subsequent peripheral and central sensitization [21, 22]. However, for the changes that have already occurred in the peripheral and central nervous systems, PRF therapy relies on modulation at the microscopic or subcellular level [23], such as synaptic plasticity modulation [24], inhibiting ectopic spontaneous discharges and enhancing adrenergic and serotoninergic descending inhibitory pathways [25], to reverse the sensitization process, and this process is relatively lengthy. In contrast, nerve block may offer rapid analgesic effects while also dilating blood vessels, improving microcirculation, and preventing the development of PHN [7]. However, the specific mechanism of Gasserian ganglion block is not yet clear; it may involve the interruption of the vicious cycle of pain, block of the sympathetic nerves, dilation of the nutritional blood vessels in the affected area, and improvement of the nutritional status of nerves [26]. Trigeminal ganglion block provides a sedative effect, reducing excessive excitation of the sympathetic nervous system and neurotransmitter release [27]. Lidocaine rapidly induces a pain-free state in the affected area, leading to muscle relaxation, vasodilation, and improvement of microcirculation [28]. Dexamethasone has anti-inflammatory, analgesic, and immunosuppressive effects and extends the duration of effectiveness when used in combination with local anesthesia [29].

Intervention timing is an important concern for pain physicians. Consensus regarding the timing of intervention for HZ and PHN has not been firmly established, but some guidelines suggest considering intervention as a second-line treatment, only opting for it when conservative treatments have failed [30]. However, early intervention may be necessary, as current analgesic treatments (including antidepressants, antiepileptic drugs, opioids, and local medications) often fail to alleviate symptoms. Kim et al. reported that early interlaminar injections performed during the acute phase of HZ (within 30 days) significantly reduced the time needed for relief from HZ-related neuralgia and the incidence of PHN compared to injections performed in the subacute stage [31]. According to the literature, the incidence of PHN is reported to be approximately 12.5%–38.4%, and it increases with age [32, 33]. Our study found that the incidence of TPHN in the acute phase group was 13.04%, significantly lower than 33.33% in the subacute phase group, and the difference was statistically significant. This suggests that HL-PRF combined block during the acute phase of HZ can effectively reduce the incidence of TPHN. A prospective controlled study [34] conducted a 5-year follow-up to compare the incidence and duration of PHN. The results indicated that performing selective nerve root block during the acute phase can reduce the incidence and shorten the duration of PHN. Our study defined the incidence of TPHN as persistent pain scores greater than 3 months after the onset of HZ lesions. This differs from previous studies [35], which defined PHN as sustained pain for 3 months after PRF treatment. We believe that our definition aligns better with the characteristics of PHN and contributes to our further research on early intervention treatments to prevent the occurrence of PHN in HZ-related neuralgia. In addition, the PSQI score of all patients significantly decreased after the procedure, and the acute phase group had lower PSQI score compared to the subacute phase group at different follow-up time points. This aligns with the trend of decreasing pain severity, indicating that HL-PRF combined block can significantly improve the sleep quality of HZ-related neuralgia. Early intervention during the acute phase yields better results.

Like a prospective randomized controlled study, we only followed the dosage of analgesic medications within the first month postoperatively. However, we found that the acute phase group, compared to the subacute phase group, could reduce the medication usage more rapidly, consistent with their pain severity. A prospective randomized controlled study found that at different time points after treatment, the postoperative VAS scores of the HL-PRF group were significantly lower than the sham surgery group [19]. In comparison with the sham surgery group, the HL-PRF group showed a significant improvement in SF-36 scores at 6 months posttreatment. In the first month posttreatment, the average dosage of pregabalin (mg/d) used by the PRF group was also significantly reduced compared to the sham group. No bleeding, infection, or other serious side effects occurred in both groups, demonstrating the safety of this HL-PRF technology.

Our study has several limitations: (1) Due to the lack of a control group and a relatively small sample size, the conclusions of the study must be approached with caution. Larger prospective randomized controlled trials are needed to explore the impact of HL-PRF combined with ganglion block on acute/subacute trigeminal neuralgia. (2) The study is a single-center study, and future multicenter clinical studies are needed to confirm the efficacy of HL-PRF combined with neural block for HZ-related trigeminal neuralgia. (3) Patients were followed up for 6 months after treatment. Future prospective studies with longer follow-up periods are necessary. (4) Our study used real-time fluoroscopy-guided puncture, ensuring safety during the procedure, with lower radiation doses compared to CT guidance, and being more cost-effective. However, in future research, a further comparison between these two imaging techniques, especially three-dimensional reconstructed images, is essential to determine the ultimately effective and safer choice.

In summary, this retrospective study indicates that the combined block of HL-PRF is an effective and safe interventional treatment method for patients with acute and subacute HZ trigeminal neuralgia. Receiving treatment during the acute phase leads to better therapeutic outcomes and helps prevent the occurrence of TPHN.

## Figures and Tables

**Table 1:** The summarized intraoperative and baseline data.

**Patients**	**Total (n = 83)**
Age (years, *x* ± SD)	69.99 ± 9.92
Gender, male (%)	43 (51.81%)
Weight	63.41 ± 10.94
Side, left (%)	30 (36.14%)
Branch affected, *n* (%)	
I	29 (34.94%)
II	11 (13.25%)
III	24 (28.92%)
I + II	8 (9.64%)
II + III	11 (13.25%)
Comorbidities, *n* (%)	
Hypertension	15 (18.07%)
Diabetes mellitus	12 (14.46%)
Coronary disease	6 (7.22%)
Stroke	4 (4.82%)
Preoperative NRS score	7.29 ± 1.41
Days after zoster onset (days)	34.92 ± 15.53
Preoperative PSQI score	19.35 ± 2.29
Preoperative dose of pregabalin (mg/d)	243.98 ± 57.19
Preoperative dose of tramadol (mg/d)	238.55 ± 48.97
Intraoperative voltage (V)	65.32 ± 10.21

*Note:* Values are presented as mean ± standard deviation.

Abbreviations: NRS, Numerical Rating Scale; PSQI, Pittsburgh Sleep Quality Index.

**Table 2:** The baseline data of the acute phase group and the subacute phase group.

**Patients**	**Acute zoster group (n = 47)**	**Subacute zoster group (n = 36)**	***p***
Age (years, *x* ± SD)	69.91 ± 11.0	70.08 ± 8.4	0.939
Gender, male (%)	23 (48.93%)	20 (55.56%)	0.550
Weight	64.11 ± 12.2	62.52 ± 9.2	0.524
Side, left (%)	18 (38.30%)	12 (33.33%)	0.641
Branch affected, *n* (%)			0.557
I	14 (29.79%)	15 (41.67%)	
II	8 (17.02%)	3 (8.33%)	
III	14 (29.79%)	10 (27.78%)	
I + II	5 (10.64%)	3 (8.33%)	
II + III	6 (12.77%)	5 (13.89%)	
Comorbidities, *n* (%)			0.481
Hypertension	10 (21.28%)	5 (13.89%)	
Diabetes mellitus	5 (10.64%)	7 (19.44%)	
Coronary disease	3 (6.38%)	3 (8.33%)	
Stroke	2 (4.26%)	2 (5.56%)	
Preoperative NRS score	7.29 ± 1.54	7.31 ± 1.24	0.927
Days after zoster onset (days)	24.21 ± 5.3	48.81 ± 13.4	—
Preoperative PSQI score	19.34 ± 1.94	19.36 ± 2.72	0.968
Preoperative dose of pregabalin (mg/d)	244.15 ± 57.39	243.75 ± 57.75	0.975
Preoperative dose of tramadol (mg/d)	234.04 ± 47.89	244.44 ± 47.89	0.344
Intraoperative voltage (V)	64.21 ± 12.32	66.56 ± 9.32	0.761

*Notes:* Values are presented as mean ± standard deviation.

Abbreviations: NRS, Numerical Rating Scale; PSQI, Pittsburgh Sleep Quality Index.

## Data Availability

The data that support the findings of this study are available from the corresponding authors upon reasonable request.
